# Interaction of the C-Terminal Peptide of Pulmonary Surfactant Protein B (SP-B) with a Bicellar Lipid Mixture Containing Anionic Lipid

**DOI:** 10.1371/journal.pone.0072248

**Published:** 2013-08-26

**Authors:** Alexander Sylvester, Lauren MacEachern, Valerie Booth, Michael R. Morrow

**Affiliations:** 1 Department of Physics & Physical Oceanography, Memorial University of Newfoundland St. John’s, Newfoundland and Labrador, Canada; 2 Department of Biochemistry, Memorial University of Newfoundland, St. John’s, Newfoundland and Labrador, Canada; Dalhousie University, Canada

## Abstract

The hydrophobic lung surfactant SP-B is essential for respiration. SP-B promotes spreading and adsorption of surfactant at the alveolar air-water interface and may facilitate connections between the surface layer and underlying lamellar reservoirs of surfactant material. SP-B_63–78_ is a cationic and amphipathic helical peptide containing the C-terminal helix of SP-B. ^2^H NMR has been used to examine the effect of SP-B_63–78_ on the phase behavior and dynamics of bicellar lipid dispersions containing the longer chain phospholipids DMPC-*d*_54_ and DMPG and the shorter chain lipid DHPC mixed with a 3∶1∶1 molar ratio. Below the gel-to-liquid crystal phase transition temperature of the longer chain components, bicellar mixtures form small, rapidly reorienting disk-like particles with shorter chain lipid components predominantly found around the highly curved particle edges. With increasing temperature, the particles coalesce into larger magnetically-oriented structures and then into more extended lamellar phases. The susceptibility of bicellar particles to coalescence and large scale reorganization makes them an interesting platform in which to study peptide-induced interactions between lipid assemblies. SP-B_63–78_ is found to lower the temperature at which the orientable phase transforms to the more extended lamellar phase. The peptide also changes the spectrum of motions contributing to quadrupole echo decay in the lamellar phase. The way in which the peptide alters interactions between bilayered micelle structures may provide some insight into some aspects of the role of full-length SP-B in maintaining a functional surfactant layer in lungs.

## Introduction

The cycling of lung volume during respiration is made possible by the presence of a surfactant layer, comprising lipids and proteins, that modifies surface tension at the air-water interface in alveoli to prevent alveolar collapse and reduce the work of breathing [1]–[5]. Approximately 10% of the surfactant material, by weight, is surfactant proteins (SP) with the remainder being lipid, predominantly phosphocholine (PC) and phosphoglycerol (PG) [6]–[8]. Four surfactant proteins are known. SP-A and SP-D are hydrophilic oligomers that contribute to host defense against pathogens [9]–[12]. SP-B and SP-C are small hydrophobic proteins that contribute to the surface activity of the surfactant layer [13], [14].

Surfactant material is secreted as multilamellar bodies by alveolar type II cells. At the alveolar air-water interface, this material forms a multilayer assembly comprising both monolayer and lamellar structures [3]–[5], [15], [16]. Formation of the surface active layer and maintenance of surfactant function requires both the incorporation of new material and the transfer of material between the monolayer and the lamellar reservoirs as surface area of the air-water interface cycles [5], [17]. These processes require the progressive reorganization of surfactant material into structures with specific topologies including interlayer contacts required for transfer of material between monolayer and lamellar environments [17], [18]. The hydrophobic surfactant proteins, which are known to promote adsorption and spreading of lipids into monolayers at the air-water interface [3], [4], [19], likely facilitate the restructuring of surfactant material into the functionally required morphology. SP-B, in particular, is essential for respiration [20]–[22].

SP-B consists of 79 residues per monomer. Each monomer has a net charge of +7 and 52% of the residues are hydrophobic [23]–[25]. There are at least four α-helical regions in each SP-B chain. The tertiary structure is stabilized by six cysteines that form three intramolecular disulfide bonds [14], [26]. A seventh cysteine promotes SP-B homo-dimerization by forming an intermolecular bond. Despite its being essential, the three-dimensional structure of SP-B is not known and the mechanism by which it enables surfactant function is not fully understood. It is likely that the combination of positive charge and hydrophobicity promotes its interaction with negative lipids in the surfactant layers and multilayers.

Transfer of material between monolayer and multilayer reservoir environments presumably requires close contact between monolayer and multilamellar reservoir surfaces and the formation of highly curved protrusions or stalk structures [4], [5], [27] at which contact can be initiated. One approach to studying the way in which hydrophobic lung surfactant proteins modify lipid organization has been to use lipids, like 1-palmitoyl-2-oleoyl phosphatidylethanolamine (POPE), with a propensity to form non-bilayer phases with highly curved surfaces [5], [28], [29]. In the absence of peptide, bilayers of POPE undergo a lamellar to inverted hexagonal phase transition above 70°C [5], [30]. In the presence of even small amounts of the lung surfactant proteins SP-B and SP-C together, POPE undergoes a lamellar to bicontinuous cubic phase at temperatures as low as 57°C [28]. It was later shown that SP-B, rather than SP-C or the combination of proteins, was primarily responsible for inducing observed transition to the bicontinuous phase [29]. The highly curved bilayer structures that connect aqueous compartments in the bicontinuous cubic phase may approximate the stalks that are thought to connect lamellar and monolayer structures in the surfactant multilayer at the air-water interface [5], [28].

In order to gain insight into the relationship between the function of SP-B and its structure, various SP-B fragments and constructs have been synthesized and investigated in both animal and *in vitro* studies [31]–[35]. Fragments that have been studied include SP-B_8–25_ and SP-B_11–25_ which contain the N-terminal helix of SP-B and SP-B_63–78_ which contains the C-terminal helix. Other peptides that have been derived from SP-B include constructs such as Mini-B, which is a concatenation of the N-terminal and C-terminal SP-B helices, and Super Mini-B (SP-B_1–25,63–78_) which also incorporates the seven-residue insertion sequence. These fragments and constructs have been found to retain substantial fractions of the activity of full-length SP-B [36], [37] and structures of SP-B_11–25_, SP-B_63–78_, and Mini-B have been determined by solution NMR [38]–[40].

Studies of the extent to which different SP-B fragments and constructs interfere with mechanical bilayer organization suggest that such peptides can perturb bilayer surface geometry [41], [42]. In studies using bilayers deposited on mica plates, SP-B_8–25_ and SP-B_63–78_ were both found to promote the formation of bilayer populations with bilayer normal directions distributed over a range of angles with respect to the mechanically-oriented bilayer normal [41], [42]. It was suggested that this might reflect a peptide-induced coupling of randomly oriented bilayers, at the hydration stage of sample preparation, that would then interfere with the ability of the bilayers to spread and flatten under the influence of the orienting mica surfaces during the stacking phase of sample preparation [42]. If so, then the observed perturbation of bilayer orientation in mechanically-constrained systems could, in effect, reflect peptide-induced interbilayer interactions during sample preparation rather than the peptide induced perturbation of already flat surfaces.

An important aspect of hydrophobic surfactant protein function that may not be modelled using mechanically-oriented bilayers is the promotion of fusion and lipid mixing between different layers, within the surfactant multilayer structure, that is implied by the flow of surfactant material between the surface layer and bilayer reservoirs during respiration. In this regard, bicellar mixture dispersions, having specific fractions of long-chain and short-chain lipids, provide an interesting alternative for the study of peptide-induced perturbation of bilayer organization because of their propensity to progressively coalesce into more extended structures on warming. The study reported here is based on such an approach.

Below the gel-to-liquid crystal transition temperature of the long-chain lipid component, bicellar mixtures form small disk particles which reorient isotropically [43]–[46]. The short-chain lipid component is preferentially located in highly curved edge environments and the longer chain lipid component is preferentially located in the more planar bicelle face. As temperature is raised through the liquid crystal-to-gel phase transition temperature of the longer component, the bicelle particles coalesce into larger structures, again with the shorter chain lipid component presumably located preferentially in curved edge environments. Based on small-angle neutron scattering (SANS) observations, it was suggested that this intermediate phase comprises worm-like or ribbon-like micelles [47], [48]. Based on geometric considerations, however, Triba and co-workers have suggested that this intermediate phase could also consist of highly perforated lamellae or transiently connected disks and that these would be difficult to distinguish experimentally from a ribbon-like phase [49], [50]. The most striking characteristic of this intermediate phase is that it can orient such that local bilayer normals are perpendicular to a magnetic field. This will, accordingly, be identified as the orientable phase. As temperature is raised further, the temperature range over which the magnetically orientable phase persists can vary from a few degrees to more than 10 degrees depending on sample composition. Above this range, there is a transition to a phase which is characteristic of more extended, and more randomly oriented, lamellae [47], [48], [51]–[53] but which also displays some dynamical differences from the multilamellar vesicle phase characteristic of a disaturated phosphatidylcholine dispersion [54], [55]. Bicellar mixture properties have been summarized in more detail elsewhere [55].

A number of factors contribute to the potential usefulness of bicellar mixture dispersions for studies of peptide-induced bilayer perturbation. In the isotropically reorienting bicelle and magnetically orientable phases, the planar surfaces may be more uniformly accessible to external perturbation than might be the case for multilamellar vesicle and mechanically supported bilayer stack samples. Because the orientable phase orients spontaneously in a magnetic field, without mechanical constraint, bilayer structures in such a phase may also be able to respond more freely to local perturbation. The component structures in the magnetically oriented bicellar dispersion phase are also bordered by highly curved edges that are rich in the short chain lipid component and do not form closed vesicles. Notwithstanding this topological difference, layering of bilayers in the magnetically oriented bicellar phase [48], [56] may resemble aspects of the multilayer surfactant structure proposed to exist at the air-water interface in lungs [17]. Furthermore, unlike the case with mechanically-oriented bilayer systems, interpretation of observations on bicellar dispersions is not complicated by issues related to sample hydration, uniformity of the observed perturbation throughout the bilayer stack, and the effects of the mechanical constraint itself on bilayer orientation and lipid chain orientational order. Mechanical orientation also limits the extent to which changes in phase behavior can be observed.

Phase transitions in bicellar dispersions can reflect the coalescence of smaller structures into more extended structures, or vice versa. Perturbation of bicellar phase behavior by a bilayer-associated protein or peptide might thus provide a way to examine how interactions between substructures in the bicellar dispersion are affected by the presence of that protein or peptide. The neuropeptide met-enkephalin, for example, has been reported to modify orientation temperatures in bicellar mixtures in different ways depending on the headgroup composition of the bicellar mixture [57]. In the case of SP-B or an SP-B fragment, peptide-induced modification of bicellar transition temperatures might reflect interactions relevant to surfactant function.

**Figure 1:**
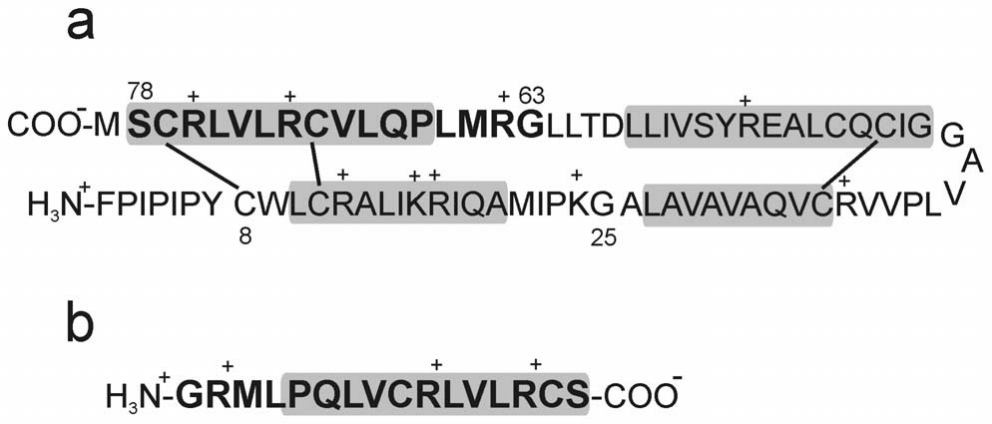
Peptide structures. (a) The primary structure of human SP-B [25] showing internal disulfide bonds. The residues included in SP-B_63–78_ are indicated in bold. The shaded boxes indicate the approximate range of residues that are expected to form helical segments. (b) The primary sequence of SP-B_63–78_.

In this work, ^2^H NMR has been used to investigate how lipid chain order, lipid dynamics, and the progressive coalescence of bicellar dispersion structures is affected by the SP-B C-terminal fragment, SP-B_63–78_. Like the full length protein, SP-B_63–78_ is positively charged (net charge of +3). The primary structures of SP-B and of SP-B_63–78_ are shown in Figure 1.

One important characteristic of the lipid component of lung surfactant is the presence of anionic lipid [4] and the bicellar mixture used for this work was a (3∶1∶1) mixture of chain perdeuterated 1,2-dimyristoyl-*sn-*glycero-3-phosphocholine (DMPC-*d*_54_), the anionic phospholipid 1,2-dimyristoyl-*sn-*glycero-3-phosphoglycerol (DMPG), and 1,2-dihexanoyl-*sn-g*lycero-3-phosphocholine (DHPC). Bicellar mixtures comprising DMPC or DMPC-*d*_54_, DMPG, and DHPC have been studied using a variety of methods including SANS[51], [56], [58]–[61], pulsed field gradient NMR [62], [63], and ^2^H or ^31^P NMR [55], [57], [64]–[66]. The properties of such mixtures are well known and the transitions occur in an accessible temperature range.

## Materials and Methods

The lipids DMPC-d_54_, DMPG, and DHPC were purchased from Avanti Polar Lipids as powders and used without further purification. SP-B_63–78_ (NH_2_-GRMLPQLVCRLVLRCS-COOH) was synthesized using solid-state methods with *O*-fluorenylmethyl-oxycarbonyl (Fmoc) chemistry and purified via HPLC as described elsewhere [39].

The bicellar lipid mixtures containing DMPC-*d*_54_/DMPG/DHPC (3∶1∶1) were prepared as described elsewhere [55]. The dry lipid mixtures, with total weights in the range of 15–25 mg, were mixed with SP-B_63–78_ to give peptide contents corresponding to 0%, 5%, 7.5% or 10% of the lipid weight. These ratios were selected to span a range of peptide fraction similar to that used in an earlier study based on mechanically-oriented bilayers [41]. The highest peptide fraction, 10%, corresponds to a lipid/protein ratio of about 30∶1. The resulting mixture was then dissolved in chloroform/methanol (2∶1 by volume). Solvent was removed by rotary evaporation at 45°C followed by exposure to vacuum for several hours. Samples were then hydrated in 100 mM HEPES buffer (pH 7) by gentle washing of the sample from the container walls. The lipid to water ratio in each sample was approximately 0.1. In order to thoroughly mix sample components and disrupt multilamellar vesicles, samples were vortexed, sonicated, and subjected to five cycles of immersion in liquid nitrogen followed by thawing at 40°C. The resulting dispersions were sealed in 8 mm diameter NMR tubes having volumes of about 400 *µ*l.

Deuterium NMR spectra were obtained using a locally assembled spectrometer based on a 9.4 T superconducting magnet. The ^2^H Larmor frequency was 61.4 MHz. Spectra were obtained using a quadrupole echo sequence [67]. The *π*/2 pulse lengths in the quadrupole echo sequence were 4–4.5 *µ*s and were separated by 35 *µ*s. Each spectrum was derived from the average of 2000–3000 transients collected with a repetition time of 0.9 s and a digitizer dwell time of 1 *µ*s. After processing to account for oversampling [68], the free induction decays that were transformed to obtain spectra had an effective dwell time of 4 *µ*s.

Quadrupole-Carr-Purcell-Meiboom-Gill (q-CPMG) echo train decay measurements can be used to gain information about relative contributions to quadrupole echo decay from “fast” and “slow” motions. In this context, fast and slow, respectively, refer to motions that modulate the orientation-dependent quadrupole interaction with correlation times that are short, relative to the characteristic ^2^H NMR time scale, (∼10^−5^ s) and motions that are too slow to contribute to motional narrowing of the ^2^H NMR spectra [55], [69]–[71]. In this study, q-CPMG was used to examine the effect of SP-B_63–78_ on the distribution of fast and slow motions. The q-CPMG pulse sequence 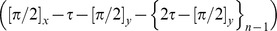 was used to generate trains of up to 40 echoes for 9 values of 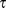 between 40 *µ*s and 500 *µ*s. For larger values of *τ*, the length of the echo train recorded was limited to 8 ms. Depending on signal, either 200 or 400 echo train transients were averaged to obtain echo amplitudes.

For small values of *τ*, decay of the q-CPMG echo train is relatively insensitive to slower motions [69]. With increasing *τ*, the effects of progressively slower motions are reintroduced. The observation of non-exponential echo train decays for larger values of *τ* is indicative of there being at least one slow motion with a correlation time that varies among different deuteron populations and fast motions that contribute similarly to echo decay for all deuterons [55], [71]. The dependence of q-CPMG echo train decays on *τ* has previously been approximated by summing signals from a limited number of deuteron populations [55], [71]. In this approximation, the quadrupole interaction of each deuteron is modulated by a slow motion that is distinct to each population and by fast motions that contribute to the echo decay for all deuterons in the same way.

In this work, q-CPMG echo train decays for each sample at 38°C have been plotted as 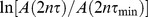 versus 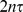 where 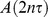 is the *n* ^th^ echo amplitude and 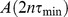 is the amplitude of the 1^st^ echo in the train obtained using the shortest 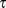. For each sample, decays were recorded for 9 values of *τ* and the resulting data set comprised 217 points. The echo train decay data for a given sample was then fit to a sum of echo train amplitudes obtained by assuming three deuteron populations with distinct slow motion correlation times and a common contribution to echo decay from fast motions. Fitting was carried out, as described elsewhere [55], using a *χ* ^2^ minimization routine within the Octave programming environment [72]. Uncertainties in the 9 parameters obtained from the fit were estimated, as described previously, from the dependence of *χ* ^2^ on the value of each parameter [55].

## Results

In this work, ^2^H NMR was used to examine the effect of the SP-B_63–78_ peptide on lipid structures in the bicellar mixture dispersions containing chain perdeuterated DMPC (DMPC-*d*_54_). For a deuteron on a specific acyl chain segment of a phospholipid undergoing axially symmetric reorientation about the normal direction of a lipid bilayer, the orientational order parameter is defined as
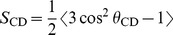
(1)where 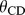 is the angle between the carbon-deuterium bond and the bilayer normal and the average is over motions that modulate the orientation-dependent quadrupole interaction on the time scale (∼10^−5 ^s) of the ^2^H NMR experiment. If the bilayer is oriented with its bilayer normal perpendicular to the applied magnetic field, the ^2^H NMR spectrum of a deuteron on a specific acyl chain segment is a doublet with a splitting of
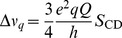
(2)where 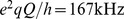 is the quadrupole coupling constant for a carbon-deuterium bond [73].

For a liquid crystalline or similarly fluid bilayer phase, 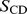 is typically smallest for acyl chain segments near the methyl end of the chain for which steric constraints on reorientation are small. Constraints on segment reorientation typically increase with distance of the segment from the bilayer centre. This is reflected in the orientational order parameter which passes through a maximum a few segments from the headgroup end of the acyl chain. The result is an orientational order parameter versus chain position profile with a characteristic plateau near the acyl chain headgroup end [73]–[76]. For oriented bilayers of chain perdeuterated phospholipid, the orientational order parameter profile can be approximated directly from the observed doublet splittings [75]. For bilayers assembled into a multilamellar morphology, the deuterons on each chain segment give rise to a Pake doublet in which the splitting of the prominent edges is given by Equation 2.

**Figure 2:**
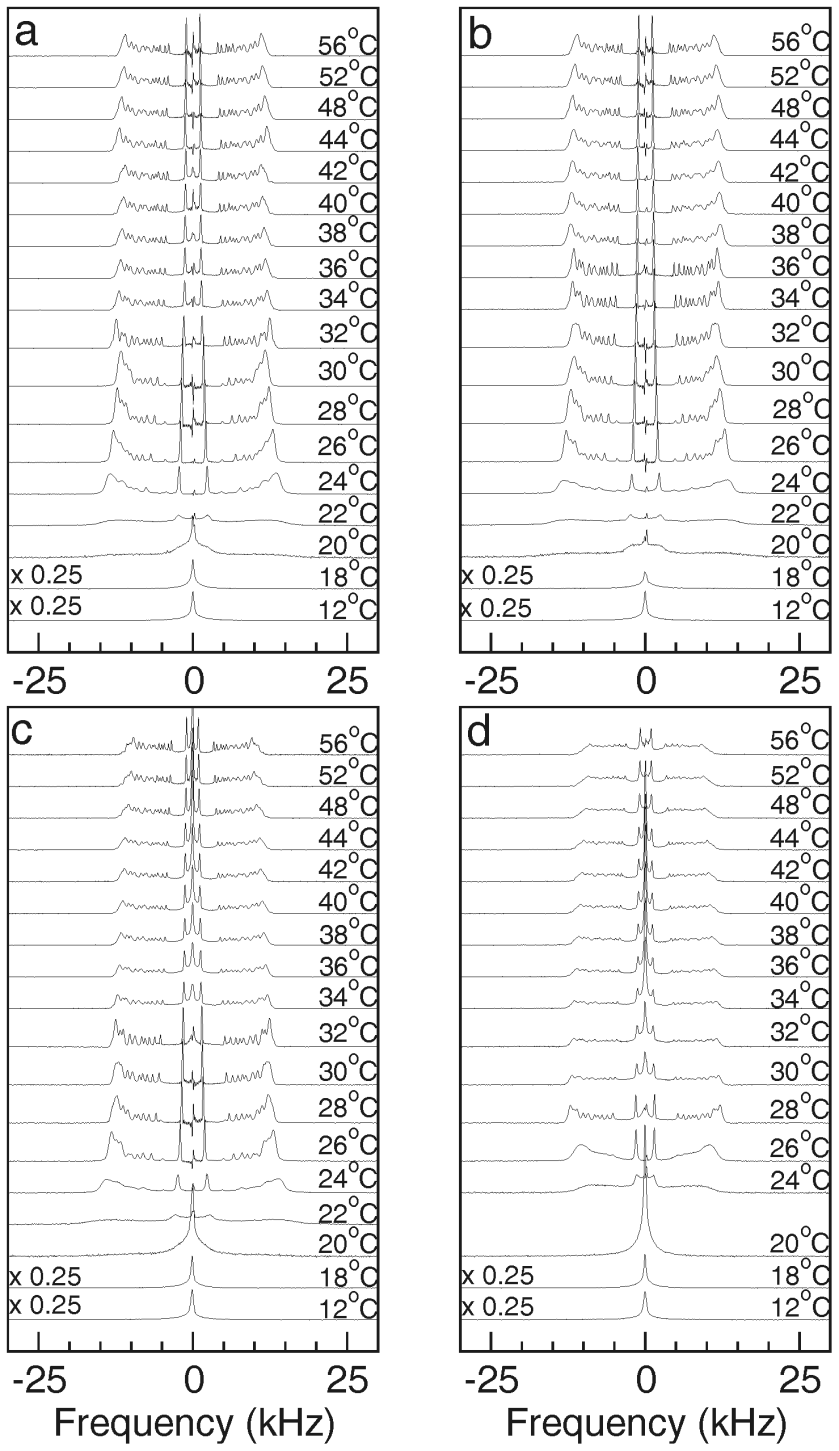
^2^H NMR spectra at selected temperatures. ^2^H NMR spectra at selected temperatures for dispersions of (a) DMPC-d_54_/DMPG/DHPC (3∶1∶1) and for DMPC-d_54_/DMPG/DHPC (3∶1∶1) with (b) 5%, (c) 7.5%, and (d) 10%, by weight, of SP-B_63–78_ present. Lipid fractions, with respect to water, are 0.1. No spectrum was recorded at 22°C for the 10% SP-B_63–78_ sample.

Figure 2 shows ^2^H NMR spectra, obtained while warming through a series of temperatures, for dispersions of DMPC-*d*_54_/DMPG/DHPC (3∶1∶1) alone and with 5%, 7.5%, and 10% SP-B_63–78_ present by weight. For DMPC-*d*_54_/DMPG/DHPC (3∶1∶1) (Figure 2a), the progression of spectral shapes, and thus phases, with increasing temperature has been reported previously [55] and is similar to what is seen for DMPC-*d*_54_/DHPC (4∶1) with no anionic lipid present [77]. Below 20°C, the spectra are narrow lines characteristic of fast, isotropic reorientation of small particles. These particles are presumed to be small bilayered micelles or bicelles. Close to the gel-to-liquid crystal transition temperatures of the long-chain lipid components (20–22°C), the particles coalesce into larger assemblies in which chain reorientation is anisotropic and not axially symmetric on the experimental time scale (∼10^−5^ s). Beginning at 24°C, the DMPC-d_54_/DMPG/DHPC (3∶1∶1) spectra become increasingly characteristic of fast, axially symmetric segment reorientation about an axis that is perpendicular to the magnetic field indicating that the sample is in the magnetically orientable phase. Around 32°C, the spectra become more characteristic of a phase with a more random distribution of bilayer normal directions. This likely reflects a further coalescence of particles into larger lamellar structures [47], [48], [51]–[53], possibly due to increased mixing of the long-chain and short-chain lipid components with increasing temperature [49], [50], [78]. The sharpness of the doublets in the lamellar phase spectra of Figure 2a is consistent with previous findings that, at low or zero concentrations of DMPG, echo decay times for the bicellar mixture lamellar phase are longer than is seen in multilamellar dispersion of DMPC-*d*_54_ at corresponding temperatures [54], [55].

^2^H NMR spectra of the bicellar dispersions containing SP-B_63–78_ display the same progression of shapes on warming the dispersions from low temperature. For 5% and 7.5% SP-B_63–78_ (Figures 2b–2c), the temperature range over which the oriented phase persists (∼24°C–32°C) is equal to or slightly larger than the corresponding temperature range in the dispersion having no peptide (∼24°C–30°C). With 10% SP-B_63–78_ (Figures 2d), the spectra show evidence of magnetic bilayer orientation over a narrower temperature range (∼26°C–28°C). Compared to the oriented phase spectra for lower peptide amounts, the spectrum of the 10% SP-B_63–78_ sample at 28°C displays a slightly less complete concentration of spectral intensity at the doublet splittings, for each deuteron, corresponding to perpendicular orientation of the bilayer normal and magnetic field. This suggests a slight perturbation of bilayer orientation. Increasing the amount of SP-B_63–78_ also appears to enhance the change in spectral shape at the transition from the oriented phase to the lamellar phase (for example, from 32°C to 34°C in Figure 2c and from 28°C to 30°C in Figure 2d) compared to the change seen at the corresponding transition in the absence of peptide (from 30°C to 32°C in Figure 2a). Increasing the concentration of DMPG in bicellar mixtures was previously found to have a similar effect [55].

**Figure 3:**
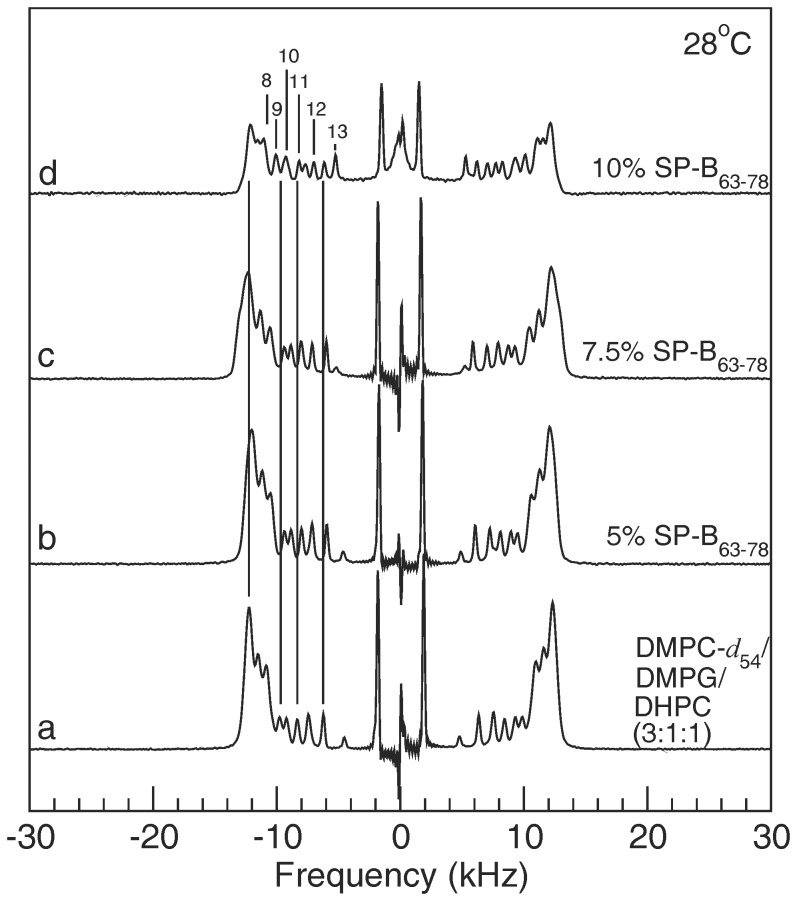
Oriented phase spectra. ^2^H NMR spectra at 28°C for dispersions of (a) DMPC-d_54_/DMPG/DHPC (3∶1∶1) and for DMPC-d_54_/DMPG/DHPC (3∶1∶1) with (b) 5%, (c) 7.5%, and (d) 10%, by weight, of SP-B_63–78_ present. Vertical lines are aids for comparison to specific splittings in the DMPC-d_54_/DMPG/DHPC (3∶1∶1) spectrum. Numerical labels on spectrum (d) indicate assignments of *sn*-1 chain carbon indices to specific doublets based on the assignment scheme presented by Petrache et al. [79].

Figure 3 shows ^2^H NMR spectra for the four samples at 28°C, the temperature at which each sample appears to be most strongly oriented in the applied magnetic field. The spectra for DMPC-d_54_/DMPG/DHPC (3∶1∶1) alone and with 5% SP-B_63–78_ (Figure 3a–3b) show the clearest evidence of orientation with doublet intensities concentrated at the quadrupole splittings corresponding to perpendicular orientation of the bilayer normal and the applied magnetic field. At 10% SP-B_63–78_ the 28°C spectrum (Figure 3d) is still predominantly characteristic of magnetic orientation but the slight enhancement of intensity between the prominent doublets, indicative of some “Pake” character within individual segment doublets, suggests a small departure from full orientation.

The vertical lines in Figure 3 are intended to facilitate comparison, between samples, of quadrupole splittings for deuterons on selected segments of the DMPC-*d*_54_ acyl chains and for the peak of the unresolved doublet feature corresponding to the orientational order parameter plateau (i.e. the feature with the largest quadrupole splitting). The numerical labels on spectrum (d) indicate the assignment of *sn*-1 chain carbon indices to specific doublets based on the assignment scheme presented by Petrache et al. [79]. As peptide concentration is raised, the quadrupole splitting of each doublet (Figures 3b–3d) decreases. This indicates a peptide-induced reduction in chain orientational order. The effect of the peptide on splittings in the plateau region is more difficult to determine due to concurrent changes in the shape of the plateau feature.

The spectrum of the sample containing 10% SP-B_63–78_ displays a very narrow doublet at the spectrum centre. This indicates that a small fraction of DMPC-*d*_54_ is undergoing nearly isotropic reorientation on the timescale of the experiment (∼10^−5^ s). This could reflect either the persistence of some small bicellar particles into the orientable phase temperature range or some migration of DMPC-*d*_54_ into the more highly curved, DHPC-rich, edge regions. The significance of component mixing to bicellar mixture phase behavior has been noted previously [49], [50], [78]. Another interesting possibility, however, is that it might reflect small regions of bilayer surface that are highly curved in response to interaction with the peptide.

For chain-perdeuterated phospholipids in an oriented bilayer, the orientational order parameter profile can be approximated by assuming that, with the exception of deuterons on the second carbon of each chain, orientational order parameters increase monotonically with decreasing carbon number along the chain [75]. The resulting profile is known as a smoothed orientational order parameter profile. This approach involves integration of a spectrum corresponding to fully oriented bilayers and is most effective for oriented samples or for samples with a completely random distribution of bilayer normal orientations such that oriented spectra can be obtained using the “de-Pakeing” transformation [75]. For bicellar mixtures, the partial orientation displayed by some spectra can preclude this approach. In this work, we have used the doublet assignment scheme described by Petrache et al. [79] to identify doublet splittings corresponding to specific carbons on the *sn*-1 acyl chain of DMPC-*d*_54_ *sn*-1 and have used these splittings to approximate orientational order parameter profiles. Deuterons on chain segments with unresolved doublets are assigned the maximum observed splitting. This does not properly capture the dependence of orientational order on carbon position in the plateau region [76] but it does facilitate direct comparison of corresponding splittings on the portion of the chain closest to the methyl end.

**Figure 4:**
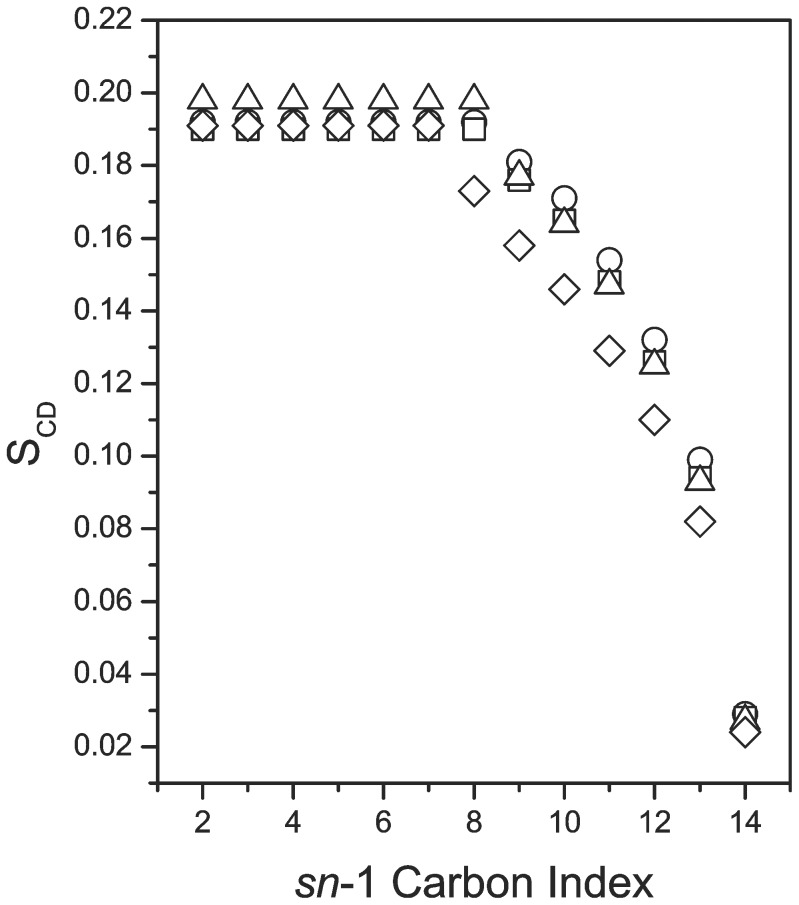
Approximate *sn*-1 chain orientational order parameter profiles at 28°C. Orientational order parameter versus *sn*-1 chain carbon index for (○) DMPC-d_54_/DMPG/DHPC (3∶1∶1) and for DMPC-d_54_/DMPG/DHPC (3∶1∶1) with (□) 5%, (Δ) 7.5%, and (◊) 10%, by weight, of SP-B_63–78_ present at 28°C.

Figure 4 compares approximate orientational order parameter profiles for the *sn*-1 chains of DMPC-*d*_54_ in the four samples at 28°C, the temperature at which the dispersions are in magnetically orientable phase. At 5% and 7.5%, the peptide reduces orientational order along the chain only slightly. At 10%, orientational order on the portion of the chain well within the bilayer interior is significantly reduced indicating that the acyl chains have more freedom to reorient. This is consistent with the peptide interacting at the bilayer surface in such a way as to slightly increase average headgroup separation. The reduction of bicelle acyl chain deuteron splittings induced by methionine-enkephalin was similarly interpreted as indicating an interfacial location for the peptide [57]. The relatively stronger perturbation at 10% peptide, compared to smaller peptide fractions, might indicate a threshold concentration beyond which the peptide interacts more strongly with bilayer surface but this cannot be confirmed without further experiments.

**Figure 5:**
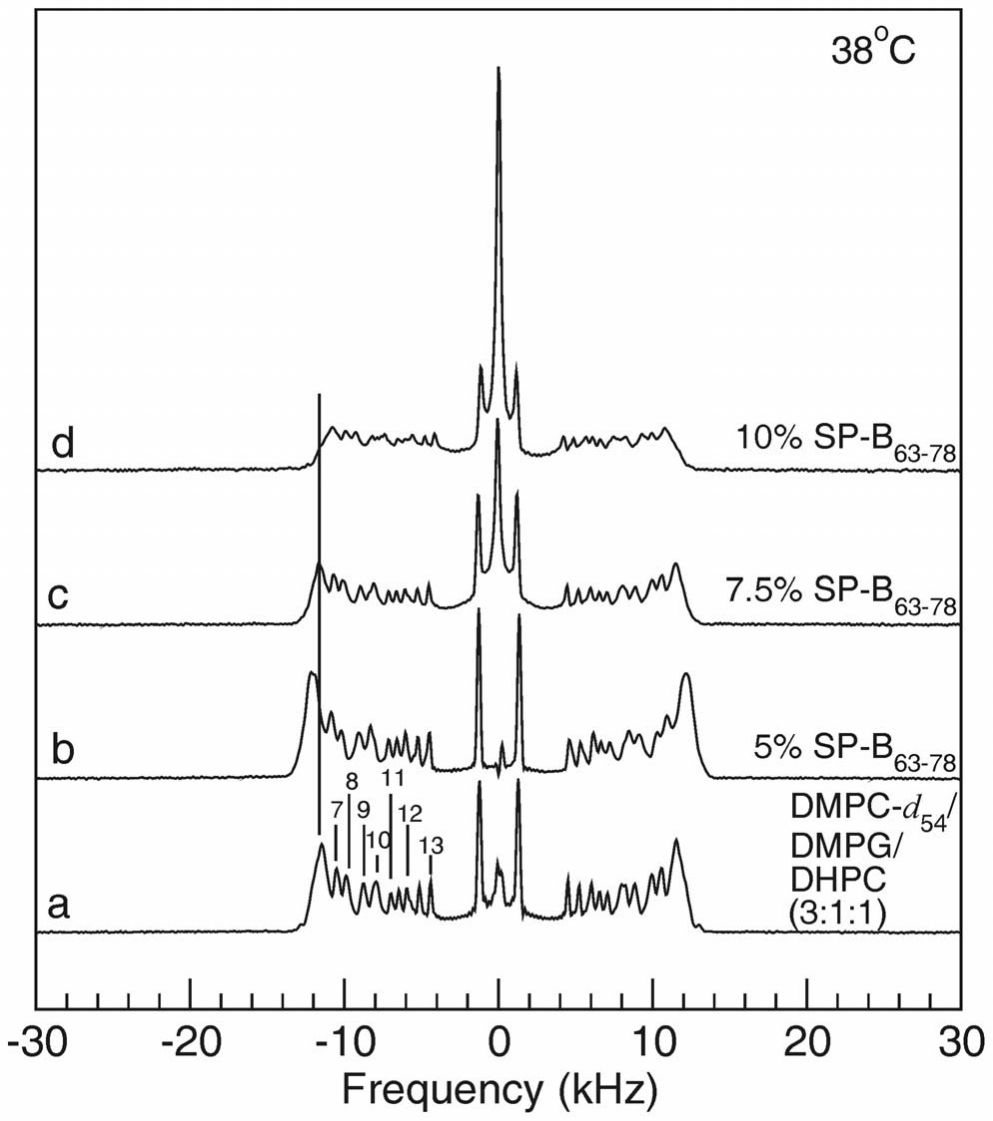
Lamellar phase spectra. ^2^H NMR spectra at 38°C for dispersions of (a) DMPC-d_54_/DMPG/DHPC (3∶1∶1) and for DMPC-d_54_/DMPG/DHPC (3∶1∶1) with (b) 5%, (c) 7.5%, and (d) 10%, by weight, of SP-B_63–78_ present. Numerical labels on spectrum (a) indicate assignments of *sn*-1 chain carbon indices to specific doublets based on the assignment scheme presented by Petrache et al. [79].

Figure 5 compares ^2^H NMR spectra for DMPC-d_54_/DMPG/DHPC (3∶1∶1) and for the peptide-containing dispersions at 38°C which is in the lamellar phase range. Again, doublets corresponding to deuterons on specific segments on the *sn*-1 chain of DMPC-*d*_54_ have been identified using the scheme described by Petrache et al. [79]. For the doublets in these spectra, intensity arising from deuterons on a given chain segment is no longer completely concentrated at the corresponding oriented-sample splitting. While the 38°C spectra for dispersions with no peptide and 5% SP-B_63–78_ (Figure 5a–5b) have some Pake character, each doublet still displays a significant concentration of intensity at the corresponding oriented phase quadrupole splitting. Despite 38°C being above the apparent oriented-to-lamellar transition for these samples, the higher temperature distribution of bilayer normal directions does not appear to be completely random for these samples. In contrast, the spectra for 7.5% and 10% SP-B_63–78_ at 38°C (Figure 5c–5d) contain no significant oriented sample component but do show a narrow central component that grows with peptide concentration. This may reflect the presence of small particles or highly curved lamellar regions but, as noted above, another possibility is that the central component might also reflect peptide-induced regions of high curvature in which lipid reorientation is more isotropic. The strong peptide concentration dependence of this feature might be consistent with such an interpretation.

**Figure 6:**
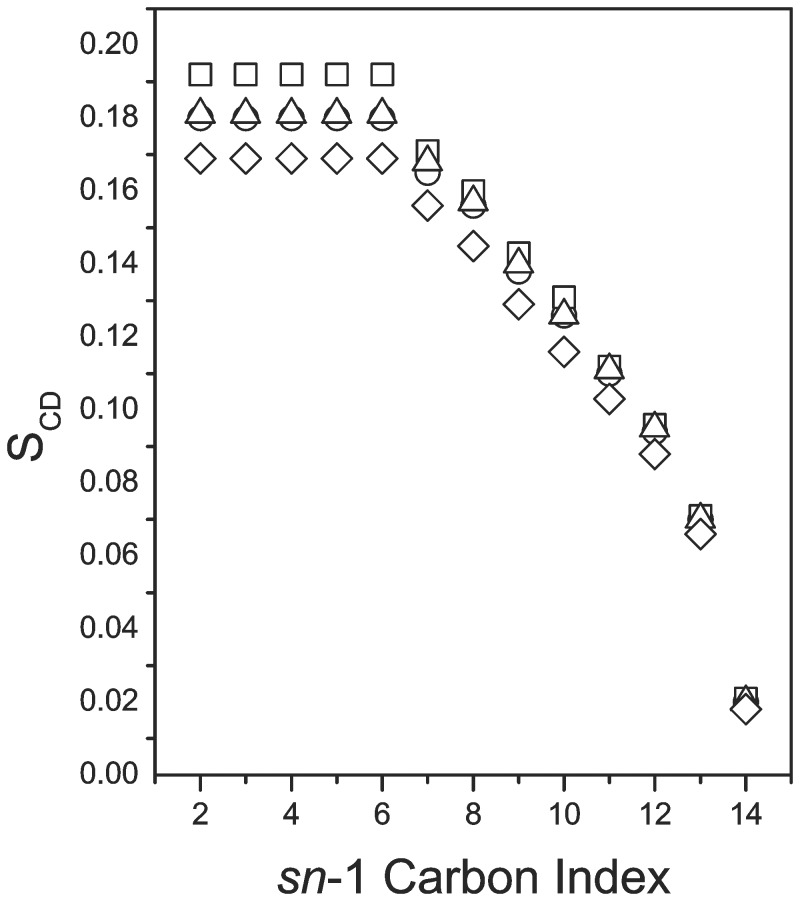
Approximate *sn*-1 chain orientational order parameter profiles at 38°C. Orientational order parameter versus *sn*-1 chain carbon index for (○) DMPC-d_54_/DMPG/DHPC (3∶1∶1) and for DMPC-d_54_/DMPG/DHPC (3∶1∶1) with (□) 5%, (Δ) 7.5%, and (◊) 10%, by weight, of SP-B_63–78_ present at 38°C.

Figure 6 shows an approximate orientational order parameter profile for the *sn*-1 chain of DMPC-*d*_54_ in the four samples at 38°C. Chain orientational order is again lowered in the sample with the largest peptide fraction but the effect is less than seen in Figure 4. This suggests that interaction with the peptide results in less perturbation of headgroup spacing in the higher temperature phase. As is seen in the orientable phase, the perturbation of chain orientational order by the peptide at fractions of 5% and 7.5% is small.

**Figure 7:**
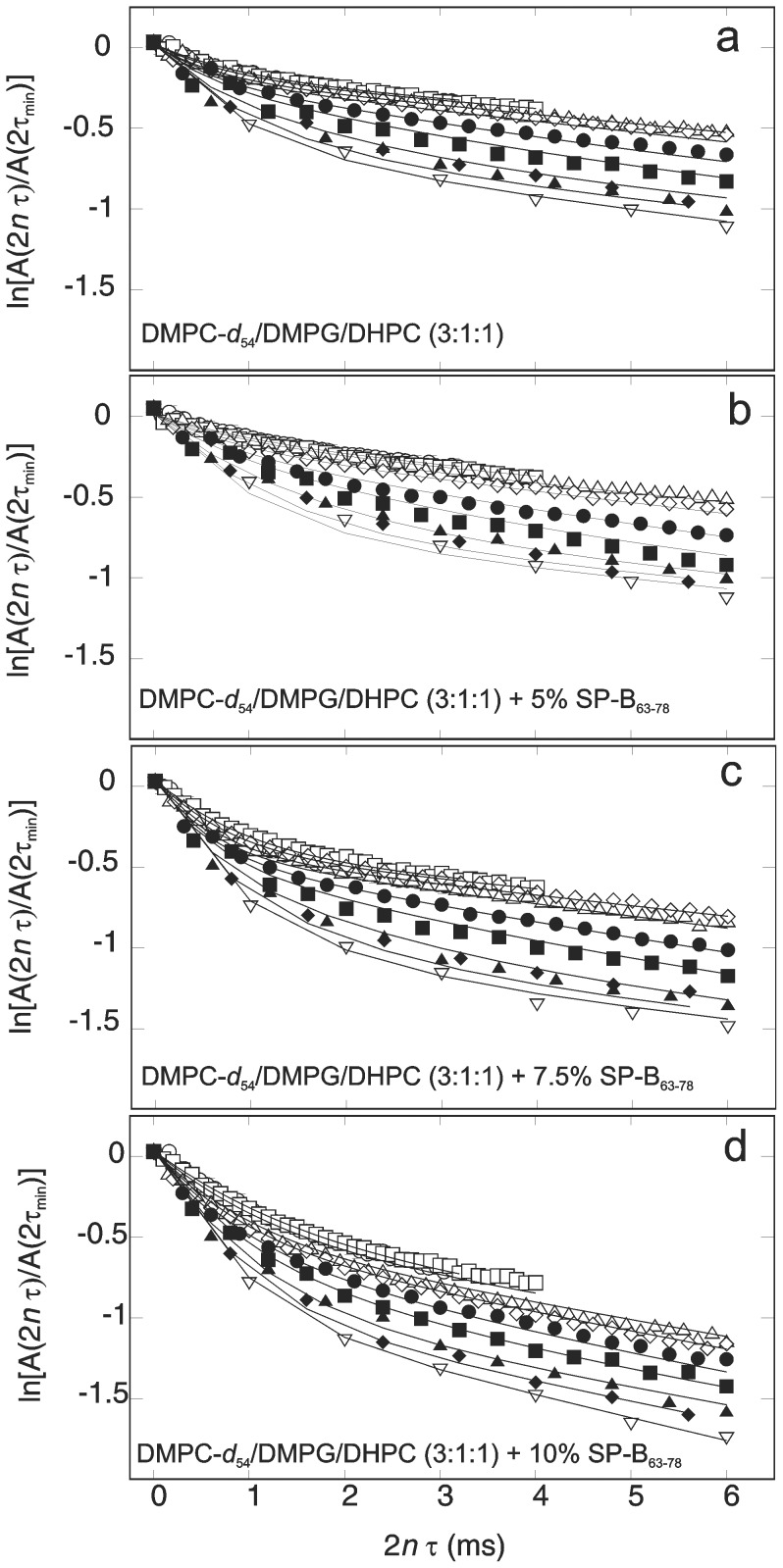
Q-CPMG echo train decays. Quadrupole Carr-Purcell-Meiboom-Gill echo-train amplitude decays at 38°C for dispersions of (a) DMPC-d_54_/DMPG/DHPC (3∶1∶1) and for DMPC-d_54_/DMPG/DHPC (3∶1∶1) with (b) 5%, (c) 7.5%, and (d) 10%, by weight, of SP-B_63–78_ present. For a given echo train with echoes separated by 2*τ*, amplitudes are plotted as 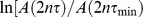 versus 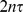 where 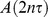 is the *n* ^th^ echo amplitude and 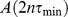 is the amplitude of the 1^st^ echo in the train obtained with the shortest 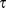. Values of 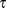 were (○) 40 *µ*s, (□) 50 *µ*s, (Δ) 75 *µ*s, (◊) 100 *µ*s, (•) 150 *µ*s, (▪) 200 *µ*s, (▴) 300 *µ*s, (♦) 400 *µ*s, and (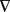) 500 *µ*s. Uncertainties are comparable to symbol sizes. Solid lines are fits to a model with three deuteron populations subject to different slow motions and a common fast reorientational motion, as described in the text.

Figure 7 shows q-CPMG echo-train decays at 38°C for DMPC-d_54_/DMPG/DHPC (3∶1∶1) alone and with 5%, 7.5%, and 10% SP-B_63–78_. At this temperature, all samples are in the lamellar phase and bilayer motions are less sensitive to small changes in temperature than might be the case closer to a transition. Echo amplitudes are recorded at times 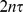 following the initial pulse of the sequence. For a given sample, each decay corresponds to a different value of *τ*. Increasing *τ* progressively reintroduces contributions, to echo decay, from motions with slower correlation times. Using q-CPMG experiments, Bloom and Sternin demonstrated that quadrupole echo decay in liquid crystalline bilayers containing deuterated phospholipid bilayers was sensitive to contributions from lipid motions that were too slow to contribute to motional narrowing of the observed ^2^H NMR spectra [69].

The interpretation of q-CPMG echo train decays in terms of bilayer motions has been described in some detail elsewhere [55]. A motion that modulates the quadrupole interaction for a given deuteron can be characterized by a correlation time, 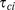, and by the second moment, 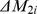, of the portion of the interaction modulated by that motion. For a q-CPMG pulse sequence that produces echoes separated by 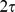, the contribution to the echo decay rate from this motion is
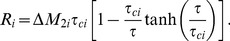
(3)


The contributions, 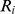, to the echo decay rate are independent of *τ* for short correlation time motions but not for slower motions (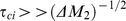) such as bilayer undulations or diffusion through regions of bilayer curvature [69].

If all deuterons experienced the same echo decay rate, the observed decays would be exponential. This is not the case for the large *τ* decays in Figure 7. For a given value of *τ*, the signal from deuterons affected by slow motions with correlation times short enough for those motions to contribute to echo decay for that pulse spacing, will decay rapidly. The remaining signal will be from deuterons affected by a superposition of even slower motions plus any fast local motions that modulate the quadrupole interaction. At large values of 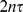, the echo decay rate for a given sample is only weakly dependent on the value of *τ*. This suggests that the contribution to echo decay from fast local motions, such as lipid wobble and rotation about the bilayer normal, is similar for all deuterons.

For liquid crystalline phospholipid bilayers, it has been found that the dependence of echo amplitude on 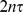 can be approximated by assuming that the deuterons can be divided into populations that have different contributions to echo decay from slow motions while having a common contribution to echo decay from fast motions [55], [71]. If 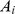 is the magnitude of the population for which the contribution to echo decay from slow motions is 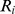, then the amplitude of the *n* ^th^ echo in a given train, in this approximation, is given by
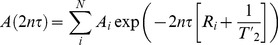
(4)where 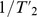 is the contribution to echo decay rate from fast motions and is assumed to be common to all deuteron populations [69].

For a given pulse spacing, the signal at large values of 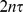, on a given echo train decay curve, comes from that fraction of the deuteron population affected by slow motions with correlation times that are too long to contribute to echo decay for that pulse spacing. For these deuterons, the echo decay rate comprises contributions only from local fast motions and is thus smaller. Increasing the pulse spacing reintroduces slow motion contributions into the echo decay of an increasing fraction of the deuteron population and the signal initially decays to a lower amplitude before the decay rate drops to the smaller value determined by common fast motions. The vertical spread, at large values of 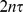, of the echo train decay curves for different pulse spacings thus reflects the distribution of correlation times for slow motions contributing to echo decay in a given sample. In Figure 6, the curves for all four samples display a similar vertical spread at large 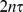 which indicates that the fraction of deuterons affected by very slow motions is roughly independent of peptide concentration. Peptide concentration does, however, affect the limiting decay rate at large values of 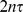. This suggests an effect on local motions with shorter correlation times. Further comparison of the bilayer dynamics responsible for the echo train decays observed in the four samples can be obtained by fitting the decays to a simple model for the distribution of slow motions across the population of deuterons.

In earlier studies, q-CPMG echo decay trains have been simulated by approximating the distribution of slow motions by a few discrete populations of deuterons with quadrupole interactions modulated by slow motions with specific correlation times [55], [71]. While somewhat arbitrary, this approach provides a basis for comparing slow motions in different samples if the discrete correlation times span an appropriate range. The solid lines in Figure 7 are simultaneous fits, to all data for a given sample, obtained by assuming that deuteron quadrupole interactions are modulated by slow motions with one of three correlation times, 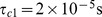, 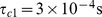, or 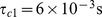. Additional details regarding the fitting procedure are provided elsewhere [55].

**Figure 8:**
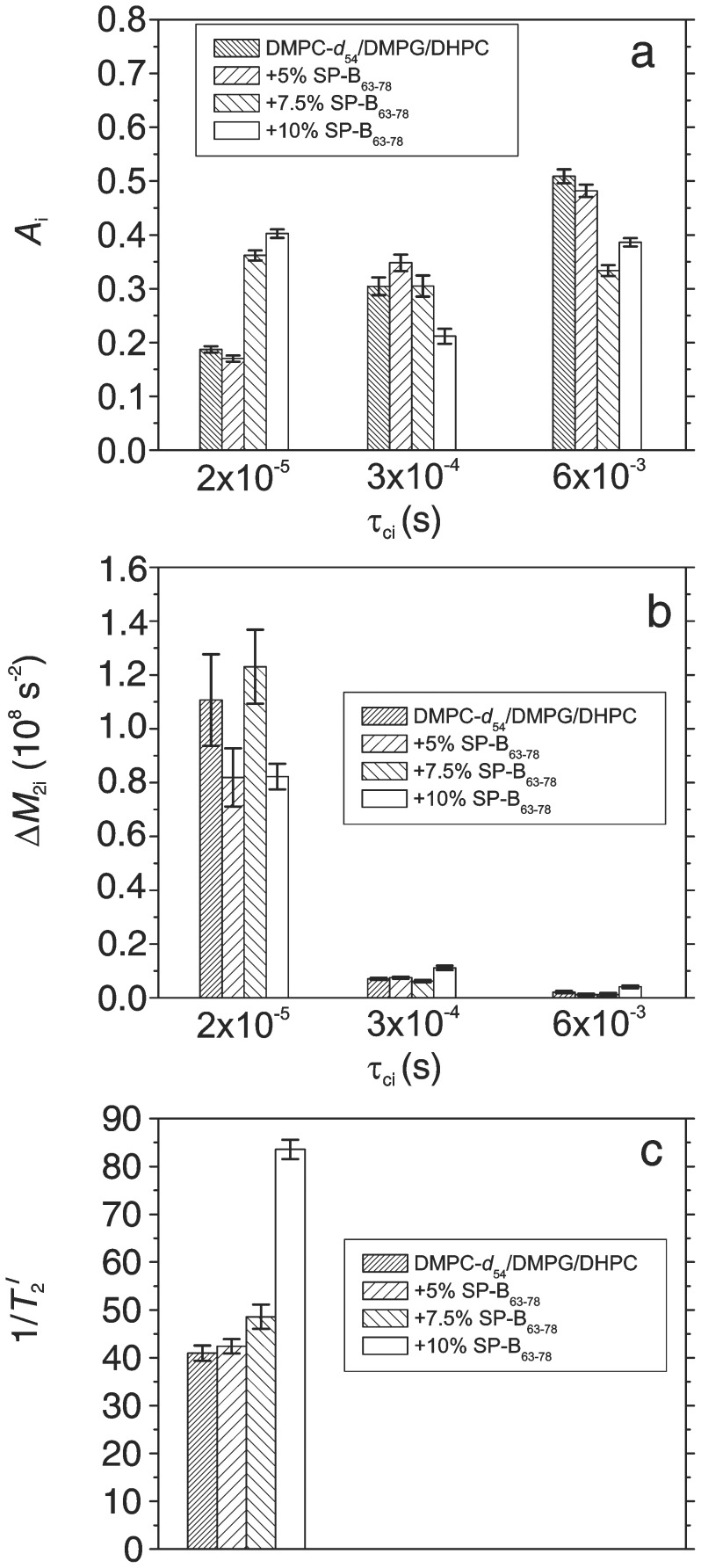
Q-CPMG decay fit parameters. Parameters corresponding to fits of the quadrupole Carr-Purcell-Meiboom-Gill echo-train amplitude decays, at 38°C, shown as solid lines in Figure 6. From left to right, respectively, the bars in each group correspond to DMPC-d_54_/DMPG/DHPC (3∶1∶1), and DMPC-d_54_/DMPG/DHPC (3∶1∶1) with 5%, 7.5%, and 10%, by weight, of SP-B_63–78_ present. Panel (a) shows relative deuteron populations in the three bins to which correlation times are constrained within the context of the model. Panel (b) shows the values of 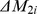 obtained from the fits for each slow motion bin. Panel (c) shows fitted contributions to echo decay rate from fast motions assumed to be common to all deuterons in the sample.

Figure 8 compares parameters obtained from fits of the q-CPMG results at 38°C to the model described above. Comparison of the relative deuteron populations (Figure 8a) suggests a shift in population from the longer correlation time bins to the shorter correlation time bin with increasing SP-B_63–78_ fraction. This suggests a shift in the spectrum of slow bilayer motions, like undulations, to shorter correlation times with increasing peptide concentration. Figure 8b shows that slow motions in the shortest correlation time bin modulate the quadrupole interaction more strongly than those in the longer correlation time bins. There does not seem to be a significant trend in second moments with peptide concentration. Figure 8c suggests that the largest effect of SP-B_63–78_ is on the contribution to echo decay rate from fast local motions. In this regime, the contribution to echo decay rate is proportional to correlation time and the second moment of that portion of the quadrupole interaction modulated by the motion. The comparison in Figure 8c thus suggests that SP-B_63–78_, particularly at a weight fraction of 10%, is either increasing the correlation time for local lipid reorientation, or increasing the amplitude of that motion. Based on the comparison of orientational order parameter profiles in Figure 4, the latter seems more likely.

## Discussion

The interaction of SP-B_63–78_ with model surfactant lipids was previously studied using mechanically oriented bilayers of POPC-*d*_31_, of POPC/POPG-*d*_31,_ or of a deuterium doped natural surfactant, bovine lipid extract surfactant (BLES) [41]. At 10% by weight, SP-B_63–78_ interfered with mechanical orientation of the bilayers and lowered the orientational order parameter along most of the deuterated acyl chains. The peptide did not induce the formation of small, isotropically tumbling lipid structures.

One possibility that has been considered is that the observed disruption of mechanical orientation induced by SP-B fragments might reflect interactions between the peptide and randomly oriented bilayers, at the hydration stage of sample preparation, that then interfered with flattening of the bilayers when orientational constraints were subsequently applied through the stacking of mica support plates [42]. Thus while the perturbation of mechanically oriented bilayers does provide some insight into interactions between SP-B fragment peptides and surfactant model bilayers, the constraints imposed by mechanical orientation may preclude observation of more dynamic perturbations of bilayer orientation or flatness by amphipathic peptides. Maintenance of the surfactant layer requires the flow of surfactant material between the layer at the air-water interface and underlying bilayer reservoirs and thus implies the formation of appropriately structured interlayer connections [5], [17]. In mechanically constrained fluid bilayers, evidence for such connections is likely difficult to identify. Mechanical orientation also makes it difficult to study peptide-induced perturbation of phase behavior.

The current work examines the interaction of SP-B_63–78_ with orientable lipid assemblies in the absence of mechanical constraints. The transitions from the isotropic to the orientable phase and from the orientable to the lamellar phase both involve the aggregation of smaller lipid assemblies into larger assemblies. The orientable phase may comprise ribbon-like micelles or perforated lamellae. Coupling of adjacent bilayers along the direction of the bilayer normal, in this phase, is likely weaker than in the higher temperature lamellar phase or in multilamellar vesicle dispersions. As a consequence, such structures may have more freedom to respond to perturbations resulting from interactions with amphipathic peptides. In particular, perturbation of the temperature at which structures in the orientable phase coalesce into more extended lamellae or multilamellar vesicles may reflect peptide-induced coupling of structures in the orientable phase.

Inspection of the spectral series in Figure 1 shows that 10% SP-B_63–78_ reduced the temperature of the oriented-to-lamellar transition in the bicellar mixture. In effect, then, the peptide appears to have facilitated aggregation of whatever structures comprise the orientable phase at a lower temperature than would been required in the absence of the peptide. The comparison of orientational order parameters in Figure 4 shows that SP-B_63–78_ reduces acyl chain orientational order in the interior of the orientable phase bilayers, presumably by interacting with the bilayer, near the hydrophobic/hydrophilic interface, in such a way as to increase the average area per lipid. In the lamellar phase, at 38°C, orientational order appears to be less sensitive to the presence of the peptide but increased peptide concentration does seem to correlate with the appearance of a small fraction of the lipid in highly curved or rapidly reorienting structures. The peptide also modifies the distribution of bilayer normal directions significantly in this phase. The q-CPMG results in the lamellar phase seem to indicate a peptide induced shift toward shorter correlation times for slow motions contributing to quadrupole echo decay and, in effect, an increase in either the correlation times or the amplitudes of fast local motions contributing to echo decay. The latter seems more likely given the effect of the peptide on slow motion correlation times. Both the spectral shape comparisons and the q-CPMG observations indicate a significant peptide-induced modification of bilayer mechanical properties in this phase.

SP-B is thought to facilitate the spreading of surfactant material and the maintenance of surfactant layer structure during respiration by promoting the formation of appropriately structured connections between adjacent layers comprising the surfactant multilayer structure [5], [17]. In the present work, SP-B_63–78_ is found to shift the equilibrium between the orientable phase and the more extended lamellar structure of the higher temperature phase. Uncertainty about the precise morphology of bilayer assemblies, in the orientable phase, makes it difficult suggest a specific mechanism but this observation suggests that the peptide does promote interaction between structures in the orientable phase. The presence of DMPG on the more planar surfaces of bicellar structure assemblies makes it likely that the peptide is interacting preferentially at bilayer surfaces and the observation that the peptide reduces DMPC-*d*_54_ chain order, and hence increases headgroup separation, supports that likelihood. Coalescence of smaller particles into more extended lamellae requires the fusing of bilayered structures and the amphipathic SP-B_63–78_ peptide may perturb the bilayered particles in such a way as to lower the energetic barrier for the formation of connections between neighbouring particles. While the results reported here suggest that the peptide interacts with bilayer surfaces, further experiments will be required to determine whether the observed perturbation of the transition from the orientable phase to the high temperature phase involves changes on the surface or at the edges of bicellar structures.

The most striking difference between the effects of SP-B_63–78_ on the DMPC-*d*_54_/DMPG/DHPC (3∶1∶1) bicellar mixtures, reported here, and its effect on mechanically oriented bilayers of POPC and POPC/POPG (7∶3), is the observation of unoriented lipid fractions in the latter case. As noted above, this may reflect an effect of the peptide on the ability of bilayers to respond to mechanical orientation during sample preparation by, effectively, forming a network of links between randomly oriented bilayers, At the transition from small isotropically reorienting particles to the orientable phase in the bicellar mixture, particles presumably have more freedom to reorient and any formation of links between particles may be less likely to result in the stabilization of a random distribution of bilayer orientations by what is, in effect, a jamming process. While similar experiments with bicelles containing unsaturated lipids might address any additional differences arising from acyl chain composition, the resulting shift in the isotropic-to-oriented transition, resulting from the lower gel-to-liquid crystal transition temperature of the unsaturated bicellar mixture component [50], would likely be a complication.

## Conclusion

^2^H NMR has been used to investigate the way in which SP-B_63–78_, the C-terminal helix of the lung surfactant protein SP-B, perturbs the phase behavior and dynamics of the bicellar mixture DPMC-*d*_54_/DMPG/DHPC (3∶1∶1). The peptide is found to lower the temperature at which structures comprising the magnetically-orientable phase of the bicellar mixture aggregate into more extended lamellae. Because the peptide is amphipathic, it is likely that it is predominantly located on the more planar faces of bilayers in the orientable phase. This is supported by the observation that higher peptide concentrations decrease acyl chain order which implies increased headgroup separation. The ability of the peptide to promote aggregation of orientable phase structures into more extended lamellar structures may thus involve the formation of protrusions and the merging of bilayer structures via stalks linking their planar faces. If so, this effect of the C-terminal SP-B helix on anionic bicellar structures might be similar to the way in which full SP-B is thought to promote transfer of material between layers in the multilayer structure formed by lung surfactant at the alveolar air-water interface [5], [17]. Perturbation of the bicellar material by SP-B_63–78_ extends into the lamellar phase where its presence results in a more random distribution of bilayer normal orientations and likely higher amplitudes for fast local reorientation of lipid molecules.

The observations reported here indicate that SP-B_63–78_ effectively softens bilayers in this system and promotes the merging of smaller lipid assemblies into more extended lamellae. This suggests that the peptide retains some of the ability of full-length SP-B to promote bilayer fusion. This property likely relates to the important roles of SP-B in promoting lung surfactant adsorption to the interface and in remodelling lung surfactant during cycles of compression and expansion. The picture of how SP-B_63–78_ interacts with the magnetically ordered and lamellar phases of this bicellar model complements that provided by earlier studies of its effect on mechanically oriented bilayers.
